# Plant Responses to Biotic Stress: Old Memories Matter

**DOI:** 10.3390/plants11010084

**Published:** 2021-12-28

**Authors:** Anirban Bhar, Amrita Chakraborty, Amit Roy

**Affiliations:** 1 Post Graduate Department of Botany, Ramakrishna Mission Vivekananda Centenary College, Rahara, Kolkata 700118, West Bengal, India; 2 EVA4.0-Unit, Faculty of Forestry and Wood Sciences, Czech University of Life Sciences, 16500 Prague, Czech Republic; chakraborty@fld.czu.cz

**Keywords:** abiotic stress, biotic interaction, epigenetic modification, histone, priming, stress memory, transgenerational immune priming

## Abstract

Plants are fascinating organisms present in most ecosystems and a model system for studying different facets of ecological interactions on Earth. In the environment, plants constantly encounter a multitude of abiotic and biotic stresses. The zero-avoidance phenomena make them more resilient to such environmental odds. Plants combat biotic stress or pathogenic ingression through a complex orchestration of intracellular signalling cascades. The plant–microbe interaction primarily relies on acquired immune response due to the absence of any specialised immunogenic cells for adaptive immune response. The generation of immune memory is mainly carried out by T cells as part of the humoral immune response in animals. Recently, prodigious advancements in our understanding of epigenetic regulations in plants invoke the “plant memory” theory afresh. Current innovations in cutting-edge genomic tools have revealed stress-associated genomic alterations and strengthened the idea of transgenerational memory in plants. In plants, stress signalling events are transferred as genomic imprints in successive generations, even without any stress. Such immunogenic priming of plants against biotic stresses is crucial for their eco-evolutionary success. However, there is limited literature capturing the current knowledge of the transgenerational memory of plants boosting biotic stress responses. In this context, the present review focuses on the general concept of memory in plants, recent advancements in this field and comprehensive implications in biotic stress tolerance with future perspectives.

## 1. Introduction

Plants have wide variations in organisational patterns, depending on their habitat and phenotypic complexity. During evolution, plants sustain in the changing environments seamlessly and have experienced multiple biotic and abiotic constraints. Biotic interventions are mainly due to pathogenesis caused by bacteria, fungi, viruses, etc. Most of these pathogenic microorganisms are also co-evolved with their plant hosts. This evolutionary arms race has continuously shaped the genetic makeup of both the host plants and their cognate pathogens. Unlike other higher eukaryotic organisms, plants hold a very high quantity of non-coding DNA in the form of repetitive and high GC-rich regions [1]. The flexible packaging of DNA with a histone core to form functional chromosome allows the nucleosome core to flexibly move along the string of DNA and permit transcriptional machinery to express prescribed portions of DNA. This transcriptional control can vastly be altered by epigenetic modification in histone proteins [2]. A great deal of pathogen-induced epigenetic control of gene regulation has been observed in many pathosystems. Recently, it has also been evidenced that these epigenetic marks can also pass on to the subsequent generation as “transgenerational memory” [3]. Pathogen-induced altered conditions are also moved to non-affected plant tissues to prepare them for future infections, called priming [4]. This priming may be temporary or permanent, depending on the intensity and frequency of infection. Sometimes, this priming also occurs by some defence-related metabolites or antimicrobial proteins [5].

Due to the sessile nature of the plants and lack of mobile defender cells, i.e., humoral immune response plants rely primarily on intricate defence signalling events, which include genetic and metabolic reprogramming in response to any pathogenic attack. Although many complex gene interaction networks have been well documented in different plants in response to various pathogens, the process of transgenerational defence signalling in plants is mainly elusive to date. The dissection of the immune memory puzzle in plants is an emerging topic of interest and capable of unveiling many unknown mysteries in plant immunology, which will enormously contribute to develop sustainable resistance in plants. In this context, the present review emphasises different aspects of the generation of immunogenic memories in plants and recent developments on the understanding of plant immunity regarding transgenerational immune memory. It also summarises challenges and future perspectives of this field of research in developing plant pathophysiology.

## 2. Stress Memory in Plants

Chester first postulated the concept of immunogenic memory in plants in 1933 and described the physiological effects of the induced plant immunity [6]. Although the concept was old, the integration of the memory concept in immunogenic reactions started during the end of the 1990s and the beginning of 2000 [7]. The actual molecular mechanism of stress memory is still being developed each day. The memory fundamentally denotes experiences of any living organism under a specialised situation, stores that information and collectively recalls the same in any future circumstances, if required. Higher animals warehouse the memory in brain cells, where it is stored and impregnated throughout their life.

Alternatively, the presence of specialised immune cells and humoral immune response in higher animals can easily store stress memory in the form of immunogenic memory cells. The development of memory in plants is questioned, as plants do not have any organised brain and nervous system. Moreover, plants do not possess any mobile defender cells, rather primarily dependent on adaptive immunity, to protect themselves against any stress factor. The development of memory against any such situations in plants is a long-debated issue, but the development of stress memory in plants in various ways has now been coming into the picture recently [8]. Although the entire mechanism of the generation of stress memory in plants is not entirely understood, the primary mechanism of genetic markings has been documented in various ways. The most well-studied area in understanding plant stress memory is epigenetic regulations. Epigenetic markings and histone modifications have a major function in gene regulation and expressional control. Many stresses, including biotic and abiotic stress factors, impose histone modifications [9]. The epigenetic control of plant stress under different biotic interventions is discussed later in this article. After successfully recognising pathogens or biotic stress, the pivotal changes inside the cellular milieu are signalling to reprogram. The inducers of such altered signal transduction vary with pathogens and associated effecter molecules. Generally, the first line of defence is initiated by altered calcium ion (Ca^2+^) induction and reactive oxygen species (ROS) generation, irrespective of pathogen types [10]. The modified redox state of the cell cytosol leads to altered transcriptional modulations and cellular signalling to attain resistance [11]. Many of these gene regulations are controlled by phosphorylation and dephosphorylations that may occur in many resistance events as memory. Phospho-proteome data in *Arabidopsis* suggested that the MYC2 and MYC3 function as the key regulators in biotic stress response in jasmonic acid (JA) signalling [12]. The role of hormonal crosstalk has a tremendous effect on biotic stress adaptation in different plants, and this has been wonderfully reviewed by Aerts et al. [13].

As mentioned earlier, cytosolic Ca^2+^ concentrations ([Ca^2+^]_Cyt_) fluctuate significantly due to several pathogens and abiotic factors. It has been evidenced that the pretreatment of attenuated biotic or abiotic stress can produce “calcium memory” within the cytosol. Such calcium signatures can induce stress-related gene expressions and enhance tolerance [14]. MAP kinase cascades also have a significant effect on biotic stress adaptations. The implication of RAS-induced extracellular signal-regulated kinase/mitogen-activated protein kinases (ERKs/MAPKs) in the plasticity of neuronal and synaptic developments, along with memory, is well evidenced in jawed animals [15]. The effect of MAPK cascades in developing memory in plants is still intangible. However, it has been observed that MAPK3, MAPK4, MAPK6 and MAPK11 are involved in innate immune response in *Arabidopsis* along with WRKY transcription factors [16]. The defence signalling events by MAPKs are intricate and have proliferous information in biotic interplay in plants. However, their connection with the generation of immune memory should be revisualised for filling the gap and solving the immune memory puzzle in plants.

## 3. Plant Immunity and Immune Memory

### 3.1. Architecture of the Plant Immune System

**Figure 1:**
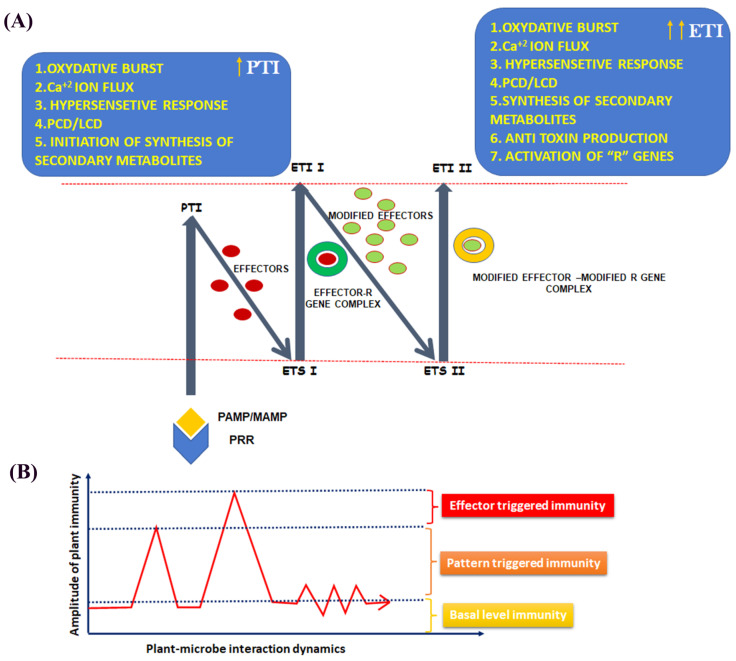
Schematic representation of plant immune response against pathogens. (**A**) Representation of the classical “zig-zag” model of the plant immunity. Pathogen-associated molecular patterns (PAMPs)/microbe-associated molecular patterns (MAMPs), when interacting with cognate host receptors (PRRs), instigate the first line of defence response called PAMP-triggered immunity (PTI). Pathogens then produce specific effector molecules or toxins that subside the PTI to the basal level, called effector-triggered susceptibility (ETS; I). Plants possess the “R” gene that produces antitoxins or inhibitors of toxins produced by the pathogens, and a second line of defence response is initiated, called effector-triggered immunity (ETI). Some pathogens can produce modified effectors to re-establish the ETS (II). This arms race continues, until one wins. (**B**). Graphical representation of the amplitude of the immune response in plants. The PTI induces sharp induction over the basal resistance response, but the amplitude of the ETI supersedes all, as it is the most specific against pathogens among all.

The plant immune system is a complex orchestration of different intracellular signalling events and is very difficult to summarise in common pathways as the reactions vary from species to species. The classical “zig-zag model” is the first proposed basic model of the plant immune system describing the arms race between hosts and pathogens [17]. Every pathogen has a signature molecule that helps hosts to identify the broad category of pathogens, called microbe-associated molecular patterns (MAMPs) or pathogen-associated molecular patterns (PAMPs), e.g., chitin present in the cell wall materials of all fungal pathogens and flg22 present in flagellated bacterial pathogens. The host plant also possesses cognate receptors for these MAMPs/PAMPs, designated as pattern recognition receptors (PRRs). PRRs first recognise pathogens by successful interaction with MAMPs/PAMPs and the very first line of defence response initiated, which is called PAMP-triggered immunity (PTI) (or MAMP-triggered immunity (MTI). The PTI response in plants is characterised by Ca^2+^ influx in cells and ROS generation [18]. The Ca^2+^ accumulation in many cases induces respiratory burst oxidase (RBOH) to generate the seamless supply of ROS that leads to oxidative burst [19]. These oxidative bursts are often associated with hypersensitive response (HR)-mediated cell death in plants, but activating inherent scavenging machinery may also lead to signature ROS for signal transduction [11,20]. The impulse of PTI initially restricts the pathogen entry, but if the pathogen survives against this armour by producing specific toxins or effectors, a subsequent downfall of an immune response happens, which is termed as effector-triggered susceptibility (ETS). Effectors are the products of the avirulence (Avr) gene of pathogens, and they are subjected to a specific target within the host body. The toxins may be of host-specific or host-non-specific types. Many host-specific toxins (HSTs) are produced by *Alternaria* sp., e.g., AM toxin I, II and III produced by *Alternaria mali*, AF toxin I, II and III by *A. alternata*, AK toxin I and II by *A. kikuchiana*, ACR toxin by *A. citri* and AAL toxin Ta and Tb by *A. alternata* f. sp. lycopersici [21]. Instead, many *Fusarium* species have produced fusaric acid as a potential mycotoxin [22]. Besides, *F. oxysporum* f. sp. *cubense* is also reported to produce beauvericin in bananas during infection [23]. To protect themselves from the deleterious effects of pathotoxins, many plants possess the “R” gene (resistance gene), which can produce antitoxins to neutralise those potential toxins. This will instigate a second wave of defence signalling called effector-triggered immunity (ETI). The ETI actuates similar events that happen during PTI, but the magnitude of defence is more than that of the PTI. On the contrary, the ETI is a more robust and specific resistance response against pathogens. Initially, it was thought that the PTI and the ETI were entirely two different sets of reactions with distinct events in the host–microbe interplay, but the continuous unveiling of physiological events of these two immune responses gradually blurred the clear distinction [24]. The evolution and diversity of the R gene in plants is enormous and an exciting field to study. Often, pathogens attack with entirely new sets of toxins to succumb to the host again, and as a result, the ETS is repeated. If the plant portrays with another cognate set of R genes against these new effectors, then the plants survive; otherwise, disease proliferates. Hence, this arms race between the host and the pathogen continues, until one successfully jeopardises the other (Figure 1). The above R–Avr interaction and the rapid development of genetic engineering technology have commissioned roles of plethora of resistance gene and utilized them in several crop improvement events [25]. Next-generation genome sequencing platforms enable the whole genome assembly of Celery (*Apium graveolens*), which demonstrated a significant number of NBS R genes within the genome [26]. Genetic polymorphism plays a crucial role in the interaction parameters of plants with their pathogenic microbes. For that reason, genetic basis of plants’ resistance now is believed to be beyond the classical “gene-for-gene” hypothesis and relies on complex gene families rather than a single gene [27].

### 3.2. Immunogenic Memory in Plants: Facts and Reality

Life first originated in water. The transformation of life from water to land came with the development of vascular plants and was designated to be one of the hallmarks of the evolution history. Land plants were originated more than 400 million years ago during the Paleozoic era [28]. Ever since, these plants have continuously been encountering various pathogenic microorganisms. The continuous battle helps plants to develop resistance against many of these pathogens. Coevolutionary interactions strongly manipulate immunogenic events in plants and promote the development of stress memory. Terrestrialisation is an impactful stress that readily affects the “evo-physio” of all land plants [29]. The physiological alterations impacted by microbes include the development of symbionts, endosymbionts, biocontrol agents and non-host resistance responses [30]. As discussed earlier, the plant–pathogen interaction largely depends on the successful interaction of specific microbes with the cognate receptors present on the host surface called “recognition reaction”. One successful recognition can only develop a disease or resistance response.

The identification and orchestration of pathogenic response by specific receptors strongly depend on plants’ memory against the repertoire of pathogenic microbes. Unlike animals, no specialised mobile defender cells have been identified in plants [30], although many signalling components may substitute immunogenic memory cells. Extracellular and intracellular receptors have contributed to the first line of immune memory. The pattern recognition receptors or PRRs, as described earlier, are responsible for the rapid recognition of pathogens and subsequent defence responses. PRRs are mostly membrane-bound and contain an outer domain protruding outside cells to interact with pathogens, called the ectodomain. Besides, there are a transmembrane domain and an intracellular domain. In most cases, this intracellular domain contains a kinase motif; hence, this type of PRRs is designated as receptor-like kinases (RLKs). In some PRRs, this intracellular domain may be absent, constituting only the ectodomain and transmembrane domain, called receptor-like proteins (RLPs). On the other hand, RLKs and RLPs are classified into many categories based on the ectodomain. The ectodomain may contain leucine-rich repeats (LRR RLKs/LRR RLPs), lysine motifs (LysMs RLKs/LysMs RLPs), lectin-like motif (LecRLKs/LecRLPs), epidermal growth factor (EGF RLKs/EGF RLPs), etc. All these receptors play a crucial role in the microbial recognition and generation of immune memory.

The first discovered PRR is Xa21, which confers resistance to bacterial blight pathogen *Xanthomonas oryzae* [31]. Another most studied PRR is Fls2 in Arabidopsis thaliana, which recognises the immunogenic epitope of bacterial flagellin protein (flg22). Both Xa21 and Fls2 are LRR-RLK-type PRRs [32]. One study has suggested that priming with Xa21 institutes basal defence signalling, particularly hormonal crosstalk and conferred resistance against a wide range of bacterial pathogens [33]. The elongation factor Tu (EF-Tu) PRR, which can recognise the elongation factor as an MAMP from bacterial pathogens, shares a common activation pathway with Fls2 in *Arabidopsis*. Although the downstream activation pathway of these PRRs is not fully understood, the involvement of a kinase, BRI1-associated receptor kinase 1 (BAK1), in this signalling event has been documented. The BAK1 mutants show diminished Fls2, and the EF-Tu mediates signalling [34]. BAK1-mediated signalling further activates MAP kinase signalling cascades to confer sustainable resistance response. One study has shown that benzothiadiazole-mediated priming in *Arabidopsis* reduces the risk of *Pseudomonas syringae* (DC3000) infection by accumulating MPK3 and MPK6 transcripts [35,36].

**Figure 2:**
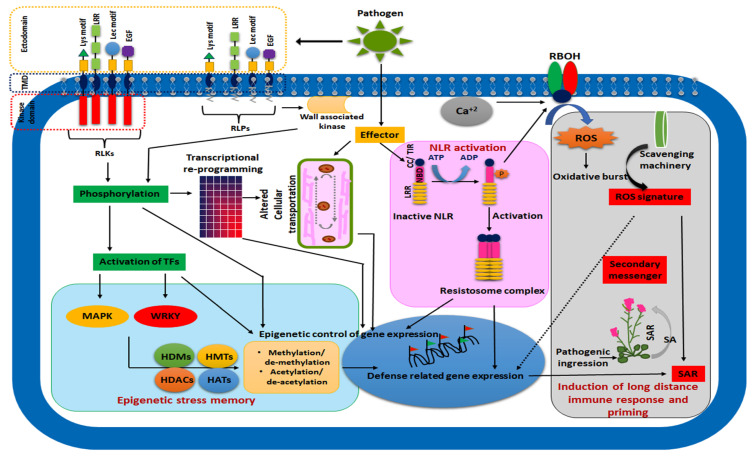
Illustration depicting the mechanistic basis of the establishment of immune memory in plants. Pathogen recognition by receptor-like kinases (RLKs), receptor-like proteins (RLPs) and intracellular NOD-like receptors (NLRs) leads to complex defence signalling and transcriptome reprogramming that induces epigenetic marks on the genome. These marks may be temporary or permanent, depending on the magnitude and frequency of the particular stress. Priming through SA and establishing SAR is a customary event for plant memory against biotic pathogens. In many cases, such biotic stress memories are transgenerational. TMD, transmembrane domain; HDM, histone demethylase; HMT, histone methyltransferase; HDAC, histone deacetylase; HAT, histone acetyltransferase; RBOH, respiratory burst oxidase; ROS, reactive oxygen species; CC/TIR, coiled-coil/toll-interleukin receptor; NBD, nucleotide-binding domain; TF, transcription factor; SA, salicylic acid; SAR, systemic acquired resistance.

On the other hand, benzothiadiazole-mediated systemic response also regulates and increases the function of Fls2 in a separate study [37], which confers that Fls2 may induce immunogenic memory by BAK1-mediated signalling that ultimately confers resistance through MPK3/MPK6-regulated cascades. It has been recently reported that EXO70B1 and EXO70B2, two subunits of the plant exocytosis complex, are involved in plasma membrane Fls2 homeostasis and signalling [38]. The ectopic expression of *Arabidopsis* L-type lectin receptor kinase-VI.2 (LecRK-VI.2) in *Nicotiana benthamiana* successfully induces priming against many bacterial pathogens. Hence, this could be used in resistance technology consortium with the Fls2 complex [39]. Oligogalacturonides (OGs) are considered a danger-associated molecular pattern (DAMP), are recognised by a different class of PRRs in the plasma membrane and activate separate signalling events. Recently, it has been observed that PCaP1, a plasma membrane-bound actin filament-associated protein, is involved in OGs recognition and also controls the late response of flagellin (flg22) signalling [40]. Like PRRs, NOD-like receptors (NLRs) are intracellular receptors for pathogens. These classes of receptors have a C-terminal LRR domain, with a central nucleotide-binding domain (NBD) and N-terminal domain. The N-terminal domain may be classified with coiled-coil (CC) or toll-interleukin1 receptor (TIR) domain. The *Arabidopsis* NLR ZAR1 produces a pentameric resistosome complex with pseudokinase RSK1 and kinase PBL2. Bacterial *Xanthomonas* effector AvrAC promotes uridylation and activates this resistosome complex to confer resistance [41]. The ADP-mediated priming of the ZAR1–RKS1–PBL2 UMP complex controls the “death switch” in NLR-mediated immune memory in plants [42]. The continuing discovery of different classes of PRRs and NLRs with their complete downstream signalling partners shapes the biology of the immunogenic memory of plants in a completely new orientation in the future (Figure 2). Additionally, phase-separated compartments in eukaryotic cells, termed as “biomolecular condensates”, is another emerging field of study in plant stress biology. These non-canonical compartments are devoid of any membranes and responsible for different important signaling events in plants including coordination of stress signals [43,44]. The role of biomolecular condensates in controlling stress memory in plants is still elusive. More research in this direction will bring new dimensions in the mechanistic behaviour of plant stress memory.

## 4. Epigenetic Regulation of Plant Memory

The long chain of DNA is organised and compressed into chromosome structures. In this compression, nucleosomes play a crucial role. Nucleosomes are histone octamers constituted of two units of each histone protein, e.g., H2A, H2B, H3 and H4, wrapped up by ~147 bp of DNA designated as the core DNA [45]. These nucleosomes are dynamic structures, allowing DNA to become available for the transcriptional machinery during gene expressions. Any changes in the topology of DNA may affect gene expression patterns. The two major pathways of epigenetic gene regulation are DNA methylation and histone modification. The methylation of cytosine residues within the DNA, which occurs mostly in promoters and non-coding regions, is a common form of DNA methylation. The de novo DNA methylation of plants is initiated by siRNAs and may occur at asymmetric (CHH) or at symmetric (CG and CHG) sites. Symmetric methylation can be maintained during DNA replication and can be inherited to progeny generations [46]. Virus-induced gene silencing (ViGS) is an interesting example of antiviral resistance response in plants, which sometimes lasts in successive generations even without an initiator virus. In such cases, mostly transcriptional silencing was noticed to be transgenerational due to the activity of methyltransferase 1 (*MET1*) in maintaining methylations in promoter regions [47]. Alternatively, the recruitment of de novo DNA methyltransferase (*DRM2*) is the prerequisite of the RNA-directed DNA methylation (RdDM) pathway, which acts as a positive feedback loop in “RNA-induced epigenetic silencing” (RNAe) [48]. The stability of these epigenetic and epigenomic marks may vary from a few days or months [9]. The passing of epigenetic memory in successive generations is still elusive. The methylome screening demonstrated that in *Arabidopsis* differential methylation positions (DMPs) are directly correlated with transgenerational acquired resistance (TAR) [49]. Recently, an interesting phenomenon has been observed in holm oak (*Quercus ilex* L.), where seedlings from the infected (*Phytophthora cinnamomi*) mother plants showed better resistance response [50]. DNA methylation and histone modification are intricately associated, as methylated DNA regions are often heterochromatic and tightly packed with histones. Different modifications on histones proteins, defined as histone post-translational modification (HPTM), can amend their packaging, wrapping characteristics, dynamics and, hence, the expression of genes [51]. In most cases, the repression in gene expression is usually carried out by the histone trimethylation in H3K9 and H3K27 positions [52]. Although not within the scope of biotic stress, vernalisation can be cited as a prime example of histone modification and its inheritance. Long cold periods are a requirement of the flowering of winter barley varieties. At low temperature, gene-activating histone modifications (lysine 4 residue of histone 3 (H3K4)) are carried out on H3 histones bound to the coding region of the barley VRN1 gene, while gene-repressing modifications (H3K27) are removed. A higher expression level of the VRN1 transcription factor, together with longer days, initiates the earing of barley. These epigenetic changes are inherited during mitotic divisions (e.g., during callus culture) but lost during meiosis, and the next generation has to be vernalised again [47,53]. In *Arabidopsis thaliana*, two homologs of human lysine-specific demethylase1-like1 (LDL1) and human lysine-specific demethylase1-like2 (LDL2) were demonstrated to control defence-related gene expression by methylation in H3K4. Mutant analyses revealed that this two-lysine-specific demethylase induces immune memory by activating WRKY transcription factors (WRKY 22/40/70) in *Arabidopsis* upon *Pseudomonas syringae* infection [54]. The complex interplay between DNA modifiers, regulating non-coding RNAs (ncRNA), chromatin remodelers and histone modifiers contributes phenotypic plasticity to sessile plants to perform better in the host–microbe interaction and the generation of stress memory [55]. Comparative proteomic analysis in *Pinus sp*. has revealed that epigenetic modifications contribute to defence signalling upon *Fusarium circinatum*, causing pine pitch canker (PPC) disease [56]. The regulation of RNA by histone modification is unknown. Nonetheless, an interesting finding has been revealed recently, where benzo-(1,2,3)-thiadiazole-7-carbothioic acid S-methyl ester (BTH)-primed *Arabidopsis thaliana* showed an increased accumulation of ARGONAUTE (AGO2 and AGO3), involved in RNA silencing. This increased transcription is due to the trimethylation of H3K4 and the acetylation of H3 in the promoter region of AGO2. This priming event conferred resistance against cucumber mosaic virus (CMV[Y]) and successfully developed resistance memory against *Pseudomonas syringae* pv. *maculicola* infection by inducing systemic acquired resistance (SAR) [57]. The epigenetic regulation of SAR was also documented in other studies. It was demonstrated that salicylic acid (SA) analogue acibenzolar S-methyl could modify the expression of defence-related genes by methylating H3K4. In this phenomenon, mostly 11 WRKY transcription factors are activated (WRKY6, WRKY11, WRKY18, WRKY22, WRKY23, WRKY26, WRKY29, WRKY31, WRKY48, WRKY53 and WRKY66) [58]. Like methylation, acetylation also has tremendous effects on biotic stress tolerance-related gene expression. Many (at least 26) putative acetylation sites are present in a single nucleosome structure, and H3 is the most prominent site for acetylation events, but recently, many acetylation sites have also been identified in H2A molecules [59,60]. The histone acetylation homeostasis is maintained by two important enzymes, histone acetyltransferases (HATs) and histone deacetylases (HDACs). It has been evidenced that one of HDACs, i.e., HDA19, in *Arabidopsis* positively regulates defence response against *Alternaria brassicicola* in JA/ethylene (ET) pathway [61]. On the contrary, the HDA19 mutant demonstrated that SA biomarkers, pathogenesis related 1 (PR1) and PR2, hyperacetylation in H3, which denoted that HDA19 helps the deacetylation and subsequent repression of these SA-related genes [62]. Nonexpressor of pathogenesis-related genes 1 (NPR1) and its paralogs NPR3 and NPR4 regulate PR1-dependent defence signalling and SAR in plants. It has been observed that NPR1 and SA largely control the function of HDAC19 to impose priming in plants [63]. In *Arabidopsis*, after pathogenic ingression, the accumulation of N-hydroxypipecolic acid (NHP) was observed, which served defence priming by inducing SAR in NPR1-dependent manner [64]. Other HDACs, such as HDA6, are also involved in JA-mediated signalling in WRKY-dependent pathways. The mutant plants exhibit higher expression patterns of SA-responsive genes (e.g., PR1, PR2, EFR, FRK1 and WRKY) to confer resistance in *Arabidopsis* against Pst DC3000 [65]. The effects of methylation and acetylation in different plant biotic responses have been reviewed in Ramirez-Prado et al. [66]. SAR is an age-old example of developing memory against biotrophic pathogens. Reduced Systemic immunity1/FLOWERING LOCUS D (RSI1; alias FLD), a known homolog of human demethylase, is required for SAR in plants. Exogenous treatment to the plants with a histone demethylase inhibitor, trans-2-phenylcyclopropylamine (2-PCPA), mimics the loss of function mutation in RSI1/FLD that promotes SAR [67]. The grafting-mediated study in *Citrus* plants showed that *Phytophthora citrophthora* induces methylation and hemi methylation, and these genomic imprints pass on root-scion combinations.

This indicates that pathogen-induced DNA marks can successfully be inherited in subsequent generations [68]. These genetic imprints largely control transcriptional circuitry in plants to combat different pathogenic attacks. A detailed dissection of transcription factor (TF) families in controlling biotic stress response in potato have revealed that epigenetic modification plays a critical role in controlling these TFs [69]. Although reports on epigenetic plant memory against biotic stress are scarce compared to those on epigenetic plant memory against abiotic stress, some excellent works have recently been documented in this regard (Table 1).

## 5. Priming vs. Memory and the Fine Line of the Difference

Any living organism inhabiting the changing environment has continuously evolved through a learning process. This learning process in plants includes thriving in adverse environmental conditions and combating different pathogenic ingressions. Environmental pressure and interaction with another organism largely shape the community structure in a best-suited fashion. Genomes of every organism can store the footprint of the past experiences, which a particular organism gathers. All of their stored genomic information and experiences are collectively called memory. The memory may be short-term or long-term, depending on the type and the intensity of the stressor. The memory formation in higher animals with organised nervous systems primarily relies on neurons and synaptic communications [82]. The promptness of the response depends on the number of neurons connecting the stimuli. In plants, the repertoire of secondary messengers, i.e., ROS, Ca^2+^, NO, SA, JA and pipecolic acid, plays a crucial role in memory response [83,84,85,86]. Short-term memory is not always associated with transcriptional reprogramming in both plants and animals. Short-term memory usually instigates the transitional expression of transcription factors that would control the set of defence-responsive genes if required [8]. This is a sort of an alert which plants and animals can receive in the intracellular milieu and prepare themselves for any future attack. This kind of situation is termed “priming”. “Memory” is usually defined for this long-term memory, which impregnate the marks in the genome in the form of acetylation, methylation, ubiquitination, SUMOylation, etc. [55,87]. Such situations can reprogram the transcriptional and translational machinery of the organism. A primed cell always can perform better than a non-primed cell against stress factors. Therefore, priming is a prerequisite for any strong memory formation in plants.

## 6. Conclusions and Future Questions

The inability to move and the lack of mobile defender cells add more complexity in defence signalling in plants. Biotic stress response primarily relies on intricate signalling pathways with sequential “switch-on” and “switch-off” mechanisms of different resistance genes. Expressional alterations, as well as the conditional expression of certain genes, are dependent on genome organization patterns and DNA methylation. These epigenetic marks on genomes stay for a while to develop stress memory in plants. Sometimes, these DNA methylations can pass generation and develop “transgenerational memory”. Different cellular signalling events can also be trained with external stress stimuli and respond accordingly, despite DNA methylations. The generation of stress memory is now evident in plants with many experimental outcomes. Still, the complete mechanistic background of memory is intangible. There are many questions to resolve and a long way to go for the complete dissection of “plant memory”. The following are some intriguing questions and outlooks associated with future research endeavours on the current topic:

1. Is the development of stress memory in plants species specific?
2. Can different pathogens make similar modifications in histones?
3. What is the actual mechanism of the transgenerational memory?
4. How stable is this transgenerational memory with and without the selection of pressure?
5. The difference between the genetics of priming and memory is still unclear.
6. The basis of the recall of memory has to be adequately resolved.

The proper inscriptions of the above scientific questions will bring new insights into the development of plant memory and enable us to utilize the same in future crop improvement strategies.

## Figures and Tables

**Table 1:** List of biotic interactions contributing to stress memory in plants.

Plants	Pathogen	Effector	Epigenetic Control	Pathway Induced	Reference
*Arabidopsis* and Apple	-	SA analogue	DNA methylation	-	Gully et al., 2019 [70]
*Sorbus aucuparia* suspension cell (SASC)	-	Yeast extract	-	Secondary metabolite induction	Yuan et al., 2021 [71]
*Arabidopsis*	-	MAMP	-	MAP kinase cascade, G3BP	Abulfaraj, 2018 [72]
*Arabidopsis*	-	-	H3K27me3 (repression) and H3K18Ac (activation)	Induction of the camalexin pathway	Zhao et al., 2021 [73]
*Arabidopsis*	*Pseudomonas syringae*	-	H3K4	LDL1 and LDL2-mediated pathway	Noh et al., 2021 [54]
Tomato (*Solanum lycopersicum*)	TSWV	-	Methylation in cytosine residue of ARF8 and miRNA167a	Auxin-mediated pathway	Werghi et al., 2021 [74]
*Arabidopsis*	*Pseudomonas syringae*	-	SDG8 mediated methylation at H3K36me3	PR1 and PR2-mediated parhway	Zhang et al., 2020 [75]
Olive (*Olea* sp.)	*Verticillium dahliae*	-	Methylation of 831 gene	-	Ramírez-Tejero et al., 2020 [76]
Tomato (*Solanum lycopersicum*)	*Pseudomonas syringae* pv*. tomato* DC3000	-	SDG8 and SDG25-induced methylation in receptor CER3 locus (H3K4 and H3K36) and in the promoter region of PR1 (H3Ac, H4Ac and H3K4me3)	SAR pathway	Chen et al., 2020 [77]
Tomato (*Solanum lycopersicum*)	*Botrytis cinerea*	-	H3K9Ac of SlyDES, SlyDOX1 and SlyLoxD	Oxylipin pathway	Crespo-Salvador et al., 2020 [78]
Rice (*Oryza sativa*)	Black Streaked Dwarf Virus (RBSDV)	-	Argonoute (OsAGO2) methylates and suppresses hexokinase (OsHXK1)	ROS-mediated pathway	Wang et al., 2021 [79]
Tobacco (*Nicotiana tabacum*)	-	SA	Epigenetic modification at H3K9, H4K20 and H4K16 of PR1a gene	SAR pathway	Lodhi et al., 2021 [80]
Common bean (*Phaseolus vulgaris*)	*Pseudomonas syringae* pv. *phaseolicola*	INA (2,6 dichloro isonicotinic acid)	Epigenetic modification of H3K4me3 and H3K36me3 of PvPR1 gene promoter	SAR pathway	Martínez-Aguilar et al., 2021 [81]

## Data Availability

The data presented in this study are available in this article only.

## Abbreviations

MYC, myelocytomatosis homologue; ERK, RAS-induced extracellular signal-regulated kinase; MAPKs, mitogen-activated protein kinases; PAMP, Pathogen-associated molecular pattern; MAMP, microbe-associated molecular pattern; PRR, pattern recognition receptor; PTI: pattern-triggered immunity; ETI, effector-triggered immunity; ETS, effector-triggered susceptibility; RLK, receptor-like kinase; RLP, receptor-like protein; NLR, NOD-like receptor; NBD, nucleotide-binding domain; CC, coiled-coil; TIR, toll-interleukin receptor.
